# Level of perioperative B-type natriuretic peptide associates with heart function after on-pump coronary artery bypass graft surgery on a beating heart

**DOI:** 10.12669/pjms.312.6189

**Published:** 2015

**Authors:** Baocai Wang, Zhaoyun Cheng, Zhenwei Ge, Bangtian Peng, Ziniu Zhao, Xiaoqiang Quan

**Affiliations:** 1 Baocai Wang, Department of Cardiovascular Surgery, Henan Provincial People’s Hospital, Henan Cardiovascular Disease Institute, Zhengzhou 450003, P.R. China; 2 Zhaoyun Cheng, Department of Cardiovascular Surgery, Henan Provincial People’s Hospital, Henan Cardiovascular Disease Institute, Zhengzhou 450003, P.R. China; 3 Zhenwei Ge, Department of Cardiovascular Surgery, Henan Provincial People’s Hospital, Henan Cardiovascular Disease Institute, Zhengzhou 450003, P.R. China; 4 Bangtian Peng, Department of Cardiovascular Surgery, Henan Provincial People’s Hospital, Henan Cardiovascular Disease Institute, Zhengzhou 450003, P.R. China; 5 Ziniu Zhao, Department of Cardiovascular Surgery, Henan Provincial People’s Hospital, Henan Cardiovascular Disease Institute, Zhengzhou 450003, P.R. China; 6 Xiaoqiang Quan, Department of Cardiovascular Surgery, Henan Provincial People’s Hospital, Henan Cardiovascular Disease Institute, Zhengzhou 450003, P.R. China

**Keywords:** B-type natriuretic peptide, Cardiac function, On-pump coronary artery bypass surgery, Perioperative

## Abstract

**Objective:**

To explore the relationship of the perioperative B-type natriuretic peptide (BNP) level with heart function among patients undergoing on-pump coronary artery bypass graft surgery on a beating heart.

**Methods:**

Total 90 patients expected to undergo coronary artery bypass graft surgery were selected and their left ventricular ejection fraction (LVEF) were examined before operation. Patients with LVEF greater than or equal to 50% were selected as the A group (n=46), and those less than 50% formed the B group (n=44). BNP levels of the patients were examined and its relationship with cardiac function was analyzed.

**Results:**

BNP levels of group A was lower than that in group B pre-and post-operatively (until 7 days after the surgery), the difference is statistically significant (p<0.05). Pearson analysis showed that the BNP level was negatively correlated with the LVEF (r = 0.767, p< 0.05). The area under the Roc curve is 0.865.

**Conclusion:**

BNP level was negatively correlated with the LVEF. Perioperative BNP level can be used as the prediction for heart function of patients with on-pump coronary artery bypass graft surgery on a beating heart.

## INTRODUCTION

Coronary artery disease (CAD), which is often the leading cause of death worldwide, is common in elderly people, and the morbidity increases every year.^1^,^2^ The patient who has CAD with left ventricular dysfunction, which occur in a high incidence, often has a poor prognosis, the clinical treatment are coronary artery bypass graft (CABG) and drugs.^3^ CABG could expand the coronary artery, ameliorate the myocardial ischemia, save the myocardium and improve the cardiac function, then prolong the life of patients. CABG is one of the most important measures for therapy of the ischemic heart diseases such as acute myocardial infarction and angina.^4^-^6^ CABG is commonly used for treating the CAD, but approximately one half of the CAD patients accompany with acute myocardial infarction or left ventricular dysfunction, who is dangerous when undergoing the on-pump CABG surgery, but this risk can be avoided when instead of off-pump CABG surgery on a beating heart.^7^,^8^ On-pump CABG surgery is widely used at present, which can effectively reduce the incidence of postoperative complications, but also may lead to myocardial instability in patient who has a low left ventricular function.^9^ On-pump CABG surgery on a beating heart is the combination of on-pump surgery and the CABG on beating heart, with the blood flow stable and low cardiac load during surgery, is conducive to revascularization and suitable for patients with serious CAD.^10^

B-type natriuretic peptide (BNP) is concerned as a diagnostic and prognostic biomarker in both ambulatory heart failure and acute coronary syndrome patients. It is reported that BNP increased in patients undergoing CABG.^11^ But the effect and mechanisms of perioperative BNP on heart function of patients undergoing on-pump CABG surgery on a beating heart has not been as clearly delineated. In the present study, we observed the levels of perioperative BNP in patients undergoing on-pump CABG surgery on a beating heart, and analyzed its relationship with the cardiac function.

## METHODS

### Study population

A total of 90 patients expected to undergo CABG surgery were recruited from the Henan Provincial People’s Hospital received from Oct 2012 to Oct 2013. The preoperative left ventricular ejection fraction (LVEF) of the patients were detected. Patients that the LVEF was greater than or equal to 50% were selected as the A group (n=46), and those less than 50% formed the B group (n=44). All patients were confirmed to have none of blood disease, liver/renal dysfunction, heart failure or respiratory insufficiency before the surgery. There were 24 men aged 21 to 68(average of 45.85 ± 16.83) and 22 women aged 22 to 65(average of 41.51 ± 18.81) in group A, and in group B, there were 21 of men aged 24 to 69(average of 44.92 ± 17.42) and 23 of women aged 20 to 66(average of 42.73 ± 19.40). The data of the two groups were comparable and had no statistically significant difference (p>0.05). This study was conducted in accordance with the declaration of Helsinki after approval from the Ethics Committee of Henan Provincial People’s Hospital. Written informed consent was obtained from all participants.

### Surgery procedure

All patients had routine examination before the operation, as well as the treatment of nitrate esters, beta-blocker and clcium antagonists. Intravenous anesthesia combined with inhalation anesthesia were used before the operation. The chest was opened by incision. The left internal mammary artery and the crura saphenous vein were heparinized by the amount of 3 m/kg, then the left internal mammary vein was picked up. Extracorporeal circulation was established between the aorta and secondary vein via cannula. The blood flow was maintained in balance when the activated clotting time was appropriate, the flow was controlled at 30-50 ml/kg/min and the blood pressure was kept at about 90/60 mmHg. The perfusion pressure was hold out using the vaso-active substance when necessary. The heart was rooted by the cardiac external fixator, the blood flow was blocked by coronary artery traction line to prevent the coronary artery bypass thrombus. The internal mammary artery and the anterior descending artery were inosculated by continuous suture, as well as the anastomosis of great saphenous vein graft far-end, vein graft and the aorta ascendens. The cessation of cardiopulmonary bypass was terminated and the protamine was used to neutralized the heparin.

### Determination of BNP and LVEF

Plasma BNP was determined by radioimmunoassay and the LVEF was measured by color doppler ultrasonic diagnostic apparatus (IU-22) (Siemens, Munich, Germany) at one hours before surgery, immediately after surgery, 6, 24, 48, 72 hours after surgery and 7 days after surgery, respectively.

### Statistical analysis

The data was expressed as mean±SD and analyzed by SPSS 17.0 (SPSS Inc, Chicago, IL, USA). Measurement data was analyzed by *t* test. Correlation analysis was performed by Pearson method. P<0.05 was considered significant.

## RESULTS

### Changes in perioperative plasma BNP concentrations

As shown in Table-I, upon surgery the levels of BNP increased, peaked at 72 hours, and then returned to preoperation level at 7 days after surgery. BNP levels of group A were lower than that of group B at any timepoint, the two groups were statistically significant difference (p<0.05).

**Table-I:** Levels of LVEF and BNP in patients undergoing CABG.

Time point	Group	BNP (pg/ml)	LVEF (%)
Preopration	A	102.75±27.05	68.96±6.53
	B	765.83±69.42	44.24±5.16
	t value	13.84	5.95
	p value	<0.01	<0.05
Immediate postoperation	A	114.86±28.95	65.75±7.03
	B	789.06±63.06	42.94±4.86
	t value	11.89	6.06
	p value	<0.01	<0.05
6 hours postoperation	A	118.96±24.06	63.96±5.85
	B	787.05±84.05	42.06±6.21
	t value	11.85	6.03
	p value	<0.01	<0.05
24 hours postoperation	A	121.64±25.05	63.06±6.23
	B	796.96±124.53	41.02±4.13
	t value	10.95	5.85
	p value	<0.01	<0.05
48 hours postoperation	A	123.74±25.04	63.02±5.85
	B	804.83±63.94	41.12±3.83
	t value	10.36	5.83
	p value	<0.01	<0.05
72 hours postoperation	A	124.85±24.95	63.01±5.09
	B	802.94±64.95	40.74±4.21
	t value	10.24	5.76
	p value	<0.01	<0.05
7 days postoperation	A	106.32±26.95	68.37±6.47
	B	658.94±53.90	48.85±5.36
	t value	10.16	5.92
	p value	<0.01	<0.05

### Correlation analysis of LVEF and BNP

**Fig.1:**
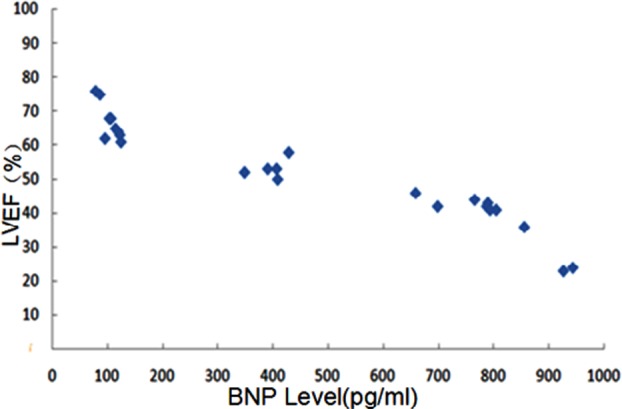
Correlation analysis of LVEF and BNP.

Correlation analysis of LVEF and BNP was performed by Pearson method. The results showed a significant negative correlation of LVEF and BNP. Patients who had high levels of BNP preserved low levels of LVEF (Fig.1), which means that plasma BNP might be a predictor of cardiac function in patients undergoing CABG.

### ROC curve

**Fig.2:**
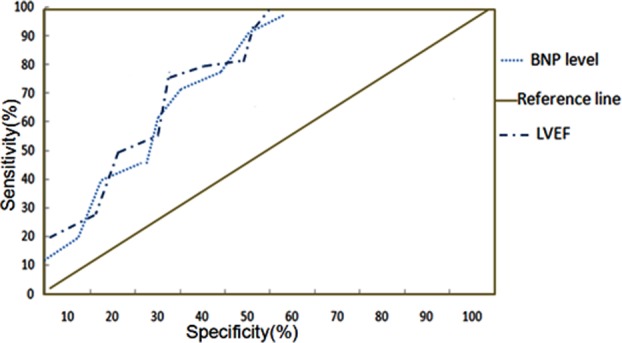
ROC curve of BNP and LVEF.

The area under the ROC curve was 0.865 (Fig.2), which was in the range of 0.7-0.9, suggesting that BNP is a good predictor for cardiac function of patients undergoing on-pump CABG surgery on a beating heart.

## DISCUSSION

Coronary artery dilatation and thrombolysis have been considered as the rapid and effective measures for treatment of severe irreversible acute coronary syndrome. But they are not suitable for multivessel coronary artery disease or patients with left main lesion, as well as patients with thrombolysis taboo or left ventricular dysfunction and patients who need the mechanical circulatory support during therapy process. On-pump CABG surgery can be carried out in the state of extracorporeal circuIation, but this surgery also has its limitations, what’s more some independent risk factors such as cardiac arrest or aortic cross clamping exist in acute coronary artery syndrome patients or severe heart function defect patients.^12^-^15^ Severe left heart failure is a risk factor of CABG, so the complications and mortality rate were high in CABG surgery for patients with poor left ventricular function, and what’s more, the on-pump surgery itself can also aggravate the myocardial injury.^16^,^17^ BNP is a natural antagonism of renin-angiotensin-aldosterone system. It has a rivalry effect for release of glucocorticoid, adrenocortical hormone, sympathetic neurotransmitter and constrictive vasoactive peptide, as well as the positive function of natriuretic and vasorelaxing, so the BNP can effectively prevent water-sodium retention and improve heart function.^18^ It has reported that BNP levels increased significantly in patients with heart failure.^19^ There are few reports about the effect of perioperative BNP on heart function of patients undergoing on-pump CABG surgery on a beating heart. The relationship of perioperative BNP and heart function is still not clear in patients undergoing on-pump CABG surgery on a beating heart. This relationship might provide a clinical basis for prediction of cardiac function in patients with on-pump CABG surgery.

In the present study, we found that in all patients upon surgery, the levels of BNP increased, peaked at 72 hours, and then returned to preoperation level at 7 days after surgery. BNP levels were relatively high in patients who preserved a relatively low LVEF levels at any time point of CABG surgery, while the a low level of BNP in patients who preserved high level of LVEF, revealed a probable negative correlation of perioperative BNP and left heart function in patients with on-pump CABG surgery, which is not consistent with the results from Hirnle.^20^ This inconsistency may be caused by the disparity of subjects investigated and detection methods. Pearson method was used to analyze the correlation of LVEF and BNP, The result suggested that LVEF and BNP were significant negative correlated in patients undergoing on–pump CABG surgery. Patients who had high levels of perioperative BNP preserved low levels of LVEF. The ROC curve also revealed that perioperative BNP is a good predictor for cardiac function of patients undergoing on-pump coronary artery bypass graft surgery on a beating heart, which was consistent with the results from Matsuura et al.^21^ The sample size in this research is small, and the mechanisms of perioperative BNP on cardiac function in patients with CABG is complex, hence a further research with larger sample size would be needed.

In summary, perioperative BNP and LVEF were negatively correlated. The perioperative BNP can predict the cardiac function in a certain extent in patients with on-pump CABG surgery on a beating heart.
